# Diversity and heterogeneity in human breast cancer adipose tissue revealed at single-nucleus resolution

**DOI:** 10.3389/fimmu.2023.1158027

**Published:** 2023-04-21

**Authors:** Lina Tang, Tingting Li, Jing Xie, Yanping Huo, Jianping Ye

**Affiliations:** ^1^ Metabolic Disease Research Center, Zhengzhou Central Hospital Affiliated to Zhengzhou University, Zhengzhou, China; ^2^ Department of Cell Biology, Key Laboratory of Cell Biology, National Health Commission of the PRC and Key Laboratory of Medical Cell Biology, Ministry of Education of the PRC, China Medical University, Shenyang, Liaoning, China; ^3^ Department of Breast Surgery, Zhengzhou Central Hospital Affiliated to Zhengzhou University, Zhengzhou, China; ^4^ Center for Advanced Medicine, College of Medicine, Zhengzhou University, Zhengzhou, China; ^5^ Research Center of Basic Medicine, Academy of Medical Sciences, Zhengzhou University, Zhengzhou, Henan, China

**Keywords:** breast cancer, adipose tissue, single-cell RNA sequencing, diversity, heterogeneity

## Abstract

**Introduction:**

There is increasing awareness of the role of adipose tissue in breast cancer occurrence and development, but no comparison of adipose adjacent to breast cancer tissues and adipose adjacent to normal breast tissues has been reported.

**Methods:**

Single-nucleus RNA sequencing (snRNA-seq) was used to analyze cancer-adjacent and normal adipose tissues from the same breast cancer patient to characterize heterogeneity. SnRNA-seq was performed on 54513 cells from six samples of normal breast adipose tissue (N) distant from the tumor and tumor-adjacent adipose tissue (T) from the three patients (all surgically resected).

**Results and discussion:**

Significant diversity was detected in cell subgroups, differentiation status and, gene expression profiles. Breast cancer induces inflammatory gene profiles in most adipose cell types, such as macrophages, endothelial cells, and adipocytes. Furthermore, breast cancer decreased lipid uptake and the lipolytic phenotype and caused a switch to lipid biosynthesis and an inflammatory state in adipocytes. The *in vivo* trajectory of adipogenesis revealed distinct transcriptional stages. Breast cancer induced reprogramming across many cell types in breast cancer adipose tissues. Cellular remodeling was investigated by alterations in cell proportions, transcriptional profiles and cell-cell interactions. Breast cancer biology and novel biomarkers and therapy targets may be exposed.

## 1 Introduction

Breast cancer is a major cause of cancer morbidity and mortality in women worldwide and has rising incidence. Obesity is a risk factor and has a negative impact on prognosis. The significant association with obesity which facilitates both incidence and progression in many tumor types, is driven by inflammatory and metabolic alterations in adipose tissue that disrupt physiological homeostasis both within local tissues and systemically (1, 2). However, underlying molecular mechanisms remain unclear. A variety of mechanisms involving, adipocytes and adipose tissue have been proposed (3, 4). The adipose tissue around the tumor is part of the tumor microenvironment and is able to regulate tumor growth through production of signaling molecules and bioenergetic substrates. The adipocyte-derived cytokines leptin, adiponectin, IL-6 and VEGF may promote tumor proliferation and transition. Bioenergetic substrates free fatty acids, cholesterol, lactic acids, glycerol and nucleotides may provide nutrition to tumor cells to support growth and mobility. It is generally believed that tumor cells may change adipocytes in the microenvironment to meet their growth demands, but the impact on adipocyte heterogeneity in breast cancer remains unknown (5–7). Adipose tissue function depends on many factors, necessitating a thorough understanding of cell types and gene expression patterns involved.

Single-cell RNA sequencing technology allows investigation of cell heterogeneity and functional status at the single-cell level to address plasticity and cellular complexity of an organ/tissue and has been used for various breast cancer and adipose cell subpopulations, but differences between breast cancer adjacent and normal breast adipose tissue from the same patient have not been assessed. In a mouse study, scRNA-seq was used to study adipocyte de-differentiation in the tumor-microenvironment (8). The results suggested that the tumor induced de-differentiation of adipocytes into myofibroblast- and macrophage-like cells caused extracellular matrix remodeling in the tumor tissue. However, the impact of the tumor on adipocytes remains to be investigated in breast cancer patients.

In the current study, adipocyte heterogeneity was compared in tumor- associated (tumor-adjacent) adipose tissue and normal (tumor-distal) adipose tissue in breast cancer patients. Single-nuclear RNA-seq (snRNA-seq) was conducted on three pairs of tumor adjacent adipose and corresponding distant normal breast adipose tissues in three postmenopausal breast cancer patients. Resident cell types were characterized to illustrate changes resulting from the proximity of breast cancer tissue. These findings give new insights into relationships between breast cancer and adipose tissue.

## 2 Methods

### 2.1 Enrollment of breast cancer patients

This study was approved by the ethics committee of Zhengzhou Center Hospital. We complied with all relevant principles of ethics, and obtained the written informed consent from all patients in this research. Three postmenopausal patients enrolled in this study were diagnosed with breast cancer by laparoscopy and pathological examination. All patients did not receive any anti-tumor treatment before surgery. Furthermore, any patients presenting with other malignant tumors were excluded. The clinical characteristics were summarized in **Supplementary Table S1**. The adipose tissue adjacent to the cancer foci is defined as the adipose tissue adjacent to the tumor (T), while with the nipple as the center point, the adipose tissue in the normal breast tissue on the opposite side of the cancer foci is defined as the normal adipose tissue (N). We collected fresh samples at the time of mastectomy and immediately placed into liquid nitrogen.

### 2.2 Tissue processing for single-nucleus suspension

Nucleus suspension preparation was performed on ice throughout. Tissue samples from patients were cut into pieces<1mm3 in 1 ml of nuclear lysis buffer (NST; 0.1% NP40, 10mM Tris-HCl, 146mM NaCl, 1mM CaCl2, 21mM MgCl2 and 40U/mL RNase inhibitor) for 7 minutes. After confirming complete nuclear lysis by trypan blue staining and microscopy, 1ml ST Wash buffer (10mM Tris-HCl, 146mM NaCl, 1mM CaCl2, 21mM MgCl2, 0.01% BSA (NEB B9000S) and 40U/mL RNase inhibitor) was added. Filter through a 40µm cell sieve (BD), transfer the filtrate to a 15mL centrifuge tube, rinse the cell sieve with ST Wash buffer, and combine the rinse with the nuclear filtrate. Centrifuge at 500g for 5 min at 4°C. Resuspend nuclei in 5ml PBS+1%BSA, wash and centrifuge, and resuspend nuclei in 100µl PBS+1%BSA. Trypan blue staining and microscopic examination.

### 2.3 10x genomics scRNA-seq

Nuclei were diluted to a concentration of 700-1200/µl with PBS + 1% BSA, and arrested *via* 10X Genomics system. According to the instruction manual of 10×Genomics Chromium Next GEM Single Cell 3′ Reagent Kits v3.1 (1000268), the machine and cDNA library were amplified. DNA library construction was performed using the Chromium™ Single Cell 3’/5’ Library Construction Kit (1000020). The constructed library was sequenced on the Illumina Nova 6000 platform using PE150 sequencing mode.

### 2.4 Gene quantitative quality control and downstream analysis

The quality control of samples was performed by using 10x genomics official software Cell Ranger, which integrates STAR software, and compared reads to the reference genome to obtain high-quality cell counts, gene counts, and genome alignment rates to assess the quality of each sample. Cells with retained cell gene counts and UMI counts within the mean ± 2 times standard deviation range, and mitochondrial gene ratios below 10% were considered high-quality cells for downstream analysis. The dimensionality reduction algorithms used in this project are PCA (Principal Components Analysis) and UMAP (Uniform Manifold Approximation and Projection) algorithms. The dimensionality reduction results based on PCA were visualized by UMAP to visualize the clustering of single-cell populations, and the clustering algorithm used SNN to finally obtain the optimal cell clustering. Cell types were annotated based on differential expression analysis for cluster-specific marker genes by using the SingleR package and HPCA reference dataset.

### 2.5 Cell types and clusters annotation

We performed cell types and clusters annotation *via* marker genes collected from public databases including Human Cell Landscape (http://bis.zju.edu.cn/HCL/), Human Cell Atlas (https://data.humancellatlas.org/), Human Protein Atlas (HPA, https://www.proteinatlas.org/humanproteome/celltype), SC2disease (http://easybioai.com/sc2disease/), Cell BLAST (https://cblast.gao-lab.org/), Cell Marker (http://biocc.hrbmu.edu.cn/CellMarker/), PanglaoDB (https://panglaodb.se/index.html), and CancerSEA (http://biocc.hrbmu.edu.cn/CancerSEA/).

### 2.6 Differential genes and enrichment analysis

Differential genes were identified using the Seurat package and the P value<0.05 and foldchange>1.5 was set as the threshold for significantly differential expression, and applied GO and KEGG enrichment analysis.

### 2.7 GSEA and GSVA

GSEA was performed with C5 GO and C2 KEGG gene sets in MSigDB (http://www.gsea-msigdb.org/gsea/msigdb) to determine the differential pathways.

The background gene set files were downloaded and organized from the KEGG database (https://www.kegg.jp/) by the use of GSEABase package (v1.44.0), and then valued the pathway activity scores on individual cells using the GSVA package (v1.30.0). Finally, calculated the difference in signaling pathway activity between different groups with LIMMA software package (v3.38.3).

### 2.8 SCENIC analysis

We conducted the single cell transcription factor network inference analysis with pySCENIC (version 0.10.3) to identify active TFs in distinct cell subtypes. The regulatory networks activity was evaluated with AUCell step. To assess the cell type specificity of each regulon, the Jensen-Shannon Divergence (JSD)-based regulon specificity score (RSS) and the Connection Specificity Index (CSI) of all regulons were calculated using the scFunctions (https://github.com/FloWuenne/scFunctions/) package.

### 2.9 ScMetabolism analysis

We quantified the metabolic activity at single-cell level *via* scMetabolism (v 0.2.1) software based on the conventional single-cell transcriptome expression matrix file, and obtained the activity scores of cells in each metabolic pathway with the use of the VISION algorithm.

### 2.10 RNA velocity analysis

Based on the output of Cell Ranger, we recounted the spliced and unsliced reads using the Python script velocy.py[43] (https://github.com/velocyto-team/velocy.py), and calculated RNA velocity values for each gene from each cell with the velocyto R package and RNA velocity vector to the UMAP two-dimensional space.

### 2.11 Trajectory analysis

We used the Monocle software package to perform machine learning based on the expression patterns of key genes, and then simulated the dynamic changes in the temporal development process. First, selected genes with a large degree of gene expression variation between cells, and performed spatial dimensionality reduction according to their expression profiles, and then constructed a minimum spanning tree (MST), and identified the longest path represented the trajectories of cells with similar transcription characteristics through the MST.

### 2.12 Cell-cell communication analysis

We systematically analyzed cell-cell communications according to ligand-receptor database with default parameters using cellphoneDB. Ligands or receptors expressed in at least 10% of cells of a certain cell type and with a P value < 0.05 were subsequently selected and we conducted ggalluvial and circlize R package to visualize communication links.

## 3 Results

### 3.1 ScRNA-seq revealed multiple cell types in breast cancer adjacent adipose tissue and normal breast adipose tissue

**Figure 1:**
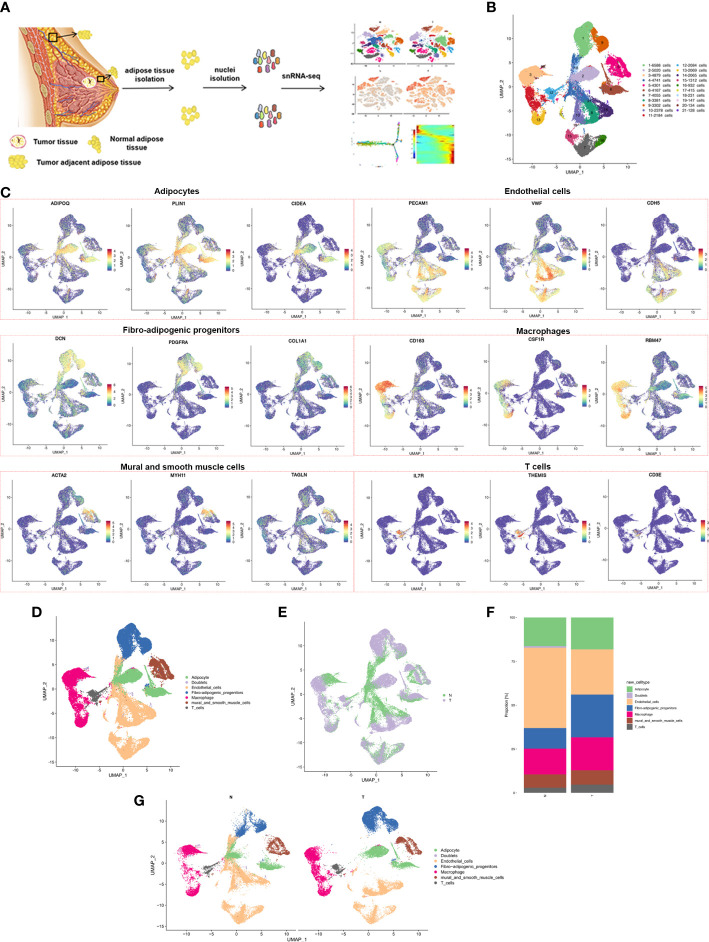
snRNA-seq of breast cancer adipose tissues. **(A)** Overview of the study workflow showing the collection and processing of samples from three patients for scRNA-seq **(B)** UMAP plot of 21 clusters of all cells from the 6 samples profiled in this study, with each cell color coded to indicate the associated subclusters and cell numbers. **(C)** Expression of cell-type-specific marker genes illustrated in UMAP plots. Canonical cell markers were used to label clusters by cell identity as represented in the UMAP plot. Cell types were classified as adipocytes, endothelial cells, fibro-adipogenic progenitors, macrophages, mural and smooth muscle cells or T cells as indicated in the legend. **(D)** UMAP plot of all cell types across 6 samples profiled in this study, with each cell color coded to indicate the associated cell types **(E)** Cells on the UMAP plot of all 6 samples were colored as originating either from N or T group. **(F)** UMAP plot of all cell types annotated in each group, with each cell color coded to indicate the associated cell types. **(G)** The relative ratio of all cell types in N and T group shown using bar plots, with each cell color coded to indicate the associated subclusters.

Three postmenopausal female patients of similar age who had received a clinical diagnosis of breast cancer were recruited. Single-cell RNA sequencing (snRNA-seq) was performed on six samples of normal breast adipose tissue (N) distant from the tumor and tumor-adjacent adipose tissue (T) from the three patients (**Figure 1A**; **Supplementary Figure S1**; **Supplementary Table S1**). A total of 65,608 single cells were isolated, and after rigorous quantitative quality control, 54513 single cells were subjected to analysis. Normal adipose yielded 26847 cells and tumor adjacent adipose 27666 cells (**Supplementary Figure S2A**). Mean reads per cell were from 31093 to 43625, and median genes per cell from 1302 to 2285 (**Supplementary Table S2**).

Adipose tissue is composed of mature adipocytes and the diverse cell-types of stromal vascular fractions (SVFs). Overlapping marker genes from the Human Cell Atlas and previously described scRNA-seq datasets of human adipose tissue were used for the performance of cluster annotation and identification of transcriptome samples. Cluster analysis divided the entire cell population into 21 clusters (**Figure 1B**; **Supplementary Figure S2B**), including fibro-adipogenic progenitors (FAPs) (cluster 1,9), adipocytes (cluster 2, 6, 18), macrophages (cluster 3, 11, 13), endothelial cells (cluster 4, 7, 8, 10, 14, 15, 16, 17, 19), mural and smooth muscle cells (cluster 5), T cells (cluster 12) and doublets (cluster 20 and 21), expressing both preadipocyte and endothelial cell markers) (**Figures 1C, D**). The cell populations contained cells from both N and T samples, indicating a common cell lineage, rather than a patient-specific sample (**Figure 1E**). A marked decrease in the relative proportion of endothelial cells and an increased proportion of macrophage and T cells in the T group was found (**Figures 1F, G**). Proportions of all 21 clusters and cell types in each patient are shown in **Supplementary Figures S2C, S2D**. In summary, snRNA-seq revealed the cellular complexity of breast adipose tissues.

### 3.2 ScRNA-seq of adipose tissues revealed distinct subpopulations of macrophage

**Figure 2:**
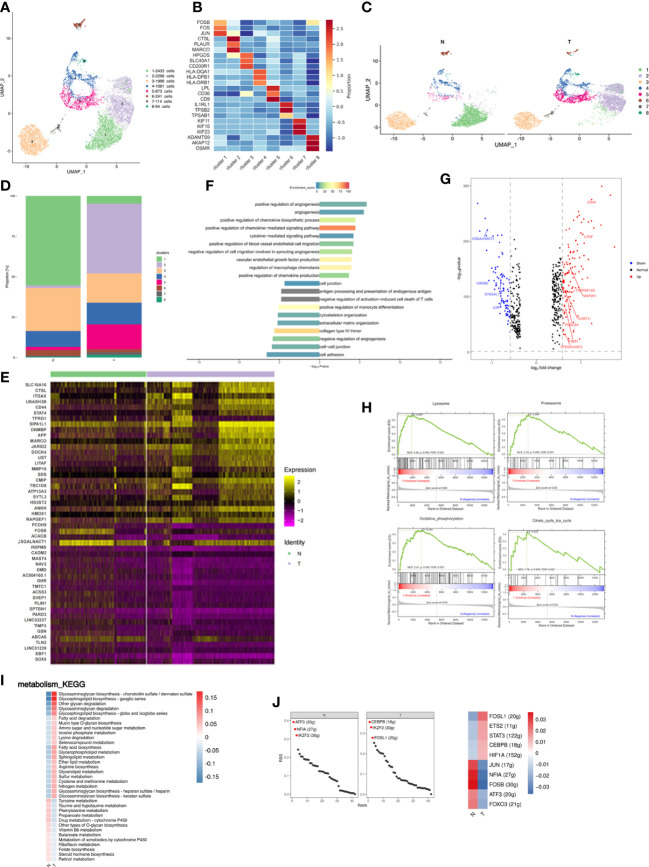
Breast cancer remodels the macrophage composition in adipose tissues. **(A)** UMAP plot of 8 clusters of macrophages from the 6 samples profiled in this study, with each cell color coded to indicate the associated subclusters and cell numbers. **(B)** Heatmap showing the expression of macrophage subpopulation marker genes in each cluster. The color changes from blue to red, indicating a low to high fold change. **(C)** UMAP plot of all macrophage subpopulations annotated in each group, with each cell color coded to indicate the associated subclusters. **(D)** The relative ratio of macrophages in N and T group shown using bar plots, with each cell color coded to indicate the associated subclusters. **(E)** Heatmap showing the top 25 differential expression genes (up- or down-regulated). The color changes from blue to red, indicating a low to high fold change. **(F)** Barplot showing the significance levels of the top 10 most significantly enriched pathways in all DEGs. The left side of the coordinate axis represents the enriched functional pathway of down-regulated genes; The right side of the coordinate axis represents the enriched functional pathway of up-regulated genes. **(G)** Expression of genes (N versus T) involving in inflammatory (GO:0006954) in macrophages. **(H)** The top 4 GSEA analysis of MARCO^+^ macrophages (cluster 2). **(I)** Heatmap indicating the enrichment of metabolism signaling pathway on all macrophages in N and T group. **(J)** The regulon specificity score (RSS) ranking plot of top 3 TFs in N and T group. The abscissa represents the ranking, and the ordinate represents the RSS score. The regulator with higher RSS may be related to the specificity of this cell group (upper). Heatmap showing the regulon activity in N and T group. Rows represent different regulons, and columns represent different cell populations. The color changes from blue to red, indicating a low to high RSS specificity score. The higher the RSS score, the stronger the specificity of regulon in this cell group (lower). The number of target genes shown in brackets.

Macrophages are the most common infiltrating immune cells in breast cancer and have both anti- and pro-tumor roles. Unsupervised macrophage subpopulation identified 8 stable clusters with unique signature genes (**Figure 2A**). Polarization phenotypic analysis failed to classify classical M1 or M2 signatures (data not shown), suggesting increased complexity of tumor-associated macrophages (TAMs) consistent with previous reports (9). Macrophage subpopulations were classified as follows. Cluster 1 exhibited high expression of AP-1 family transcription factors (expressing e.g., JUN, FOSB and FOS) which have been implicated in cell proliferation, differentiation, apoptosis, and oncogenic transformation (10). Cluster 2 may consist of pro-tumor macrophages with the specific expression of MARCO and PLAUR (expressing e.g., CTSL, MARCO and PLAUR) and the proportion was significantly increased compared with N group. MARCO is reputed to be a marker of a TAM subset associated with poor prognosis in human cancers (11–13). PLAUR may affect many normal and pathological processes related to cell-surface plasminogen activation and local degradation of the extracellular matrix (14). The activation of plasminogen and extracellular matrix degradation mediated by PLAUR are important causes of tumor metastasis (15). Overexpression of PLAUR has been observed in many cancers and is usually associated with poor survival and prognosis (16–18). Cluster 3 showed high expression of inflammatory genes (expressing e.g., HPGDS, SLC40A1 and CD200R1). Cluster 4 expressed antigen presentation associated genes, consistent with the gene signatures of dendritic cells previously reported (expressing e.g., HLA-DQA1, HLA-DPB1, HLA-DRB1 and HLA-DRA). Cluster 5 was revealed to have a transcriptional signature associated with lipid metabolism and phagocytosis (expressing e.g., LPL, CD36 and CD9), consistent with lipid-associated macrophages (LAMs) that clear dead adipocytes and lipids and have a proinflammatory phenotype (19–22). Cluster 6 may be mast cells (expressing e.g., IL1RL1, TPSB2 and TPSAB1). Cluster 7 expressed genes related to cellular proliferation and cell cycle (expressing e.g., KIF11, KIF15 and KIF23). Cluster 8 expressed genes associated with extracellular matrix remodeling and deposition (expressing e.g., ADAMTS9, OSMR and AKAP12) (**Figure 2B**; **Supplementary Table S3**). Chen et al. identified a pro-tumor subpopulation of macrophages characterized by MARCO, a mesenchymal pro-tumor marker in GBM (12). Previously published results demonstrated that the expression of MARCO correlated with expression of M2 markers expressed by tumor-promoting macrophages and EMT-metastasis-driving gene signatures in breast cancer, and antibody targeting of MARCO-expression TAMs blocked tumor growth and metastasis (23, 24). PLAUR plays a crucial role in extracellular matrix degradation and tissue remodeling, which regulates tumor invasion and metastasis, a pivotal characteristic of malignant tumors (17, 25). In addition, some studies have indicated that tumoral and macrophage PLAUR can promote tumor invasiveness and that macrophages can increase the expression of PLAUR in tumor cells (26, 27). These results suggested that PLAUR may be involved in tumor immunity and prompted us to explore further. The current data identified the high level of MARCO^+^PLAUR^+^ macrophages in adipose tissue adjacent to tumors at single cell level, indicating MARCO^+^PLAUR^+^ macrophages were related to the progression of breast cancer. Cluster 5 was very similar to the previously reported lipid-associated macrophages (LAM), which are involved in the clearance of dead adipocytes (19, 20, 28, 29). However, although clusters 1, 3 and 7 showed high expression of LYVE1 and SELENOP (**Supplementary Figure S3A**), considered to be marker genes of perivascular macrophages, further analysis showed gene expression profiles different from those published previously (29, 30), perhaps due to the limited number of samples or organ specificity.

Relative proportions of clusters 2 and 5 increased and those of clusters 1 and 3 decreased in T samples (**Figures 2C, D**; **Supplementary Figure S3B**). Increased proportions of cluster 5 indicated that breast cancer activated the non-classical inflammatory ATM phenotype involved in lipid metabolism (19, 31). Three patients were independently analyzed to investigate whether differences in cluster proportion in N and T were significantly different. MARCO^+^PLAUR^+^ macrophages were significantly increased in T (p=0.0037) (**Supplementary Figure S3C**). Macrophage gene expression was compared between the N and T groups, and different functions found to be enriched in differentially expressed genes (DEGs), indicating immune microenvironment differences between normal breast and cancer associated adipose. Analysis of DEGs identified 125 upregulated and 92 downregulated genes in T, the top 25 of which are presented as a heatmap (**Figure 2E**). Analysis of DEGs function showed that upregulated genes were associated with angiogenesis, cytokine-mediated signaling pathway, and regulation of blood vessel endothelial cell migration. Downregulated genes were related to cytoskeleton organization, extracellular organization, and cell adhesion (**Figure 2F**). Breast cancer involves a dramatic change in macrophage and cytokine profiles, which are involved in tissue remodeling, inflammation and the development of breast cancer (32). Therefore, macrophage cytokine expression in breast cancer was investigated and genes related to cytokine production and macrophage inflammatory responses analyzed. CD44 and LITAF were identified as the cytokines most highly upregulated in T group macrophages (**Figure 2G**). CD44 has been reported to modulate macrophage recruitment and regulate adipose tissue inflammation (33, 34). LITAF interacts with STAT6B to regulate expression of inflammatory cytokines (35, 36).

Relative proportions of cluster 2 were dramatically altered by breast cancer, increasing from being almost non-existent in N to being the most abundant subpopulation of T. Gene set enrichment analysis (GSEA) of cluster 2 showed the highest enrichment scores in MARCO^+^ macrophages to be lysosome, proteasome, oxidative phosphorylation (OXPHOS) and citrate cycle (TCA cycle) (**Figure 2H**), consistent with the functions of MARCO in phagocytosis and clearance (37). Changes in OXPHOS and TCA are supported by previous reports of TAM-facilitated production of ATP *via* TCA and OXPHOS in breast cancers (38, 39). These results achieved at the single cell level.

Metabolic alterations in tumor tissue and the tumor microenvironment are crucial to tumor progression and adipose inflammatory and metabolic change disrupt physiological homeostasis both within local tissues and systemically. Metabolism-based KEGG analysis showed significant enrichment in glycosphingolipid and glycosaminoglycan metabolism and fatty acid biosynthesis in breast cancer-adjacent tissues (**Figure 2I**). Glycosphingolipid biosynthesis has been reported to modulate the pro-inflammatory phases of LPS/TLR4 activation in macrophages (40). Glycosaminoglycans interact with growth factors, growth factor receptors and cytokines to regulate cancer growth, progression, and metastasis (41). The upregulation of glycosphingolipid and glycosaminoglycan metabolism and fatty acid biosynthesis during breast cancer may induce inflammation and promote cancer progression.

Analysis of transcription factors (TFs) illuminates the gene regulatory network behind cell heterogeneity and differential transcription factor activity was analyzed by single-cell regulatory network inference and clustering (SCENIC) followed by supervised cluster analysis. N tissues had enriched ATF3 and NFIA regulons, known to repress expression of proinflammatory cytokines and chemokines, while T tissues had enriched CEBPB and FOSL1 regulons which promote the macrophage inflammatory phenotype (**Figure 2J** upper) (42–45). Evaluation of differential TF activity indicated high activity of JUN, NFIA, FOSB, ATF3, and FOXO3 in the N group and high activity of FOSL1, ETS2, STAT3, and CEBPB in the T group (**Figure 2J** lower).

In summary, macrophages were the major immune cells in breast adipose tissue and breast cancer leads to a marked increase in the proportion of MARCO^+^PLAUR^+^ and lipid-associated macrophages. Furthermore, breast cancer induced an inflammatory macrophage phenotype.

### 3.3 Influences of breast cancer on endothelial cells

**Figure 3:**
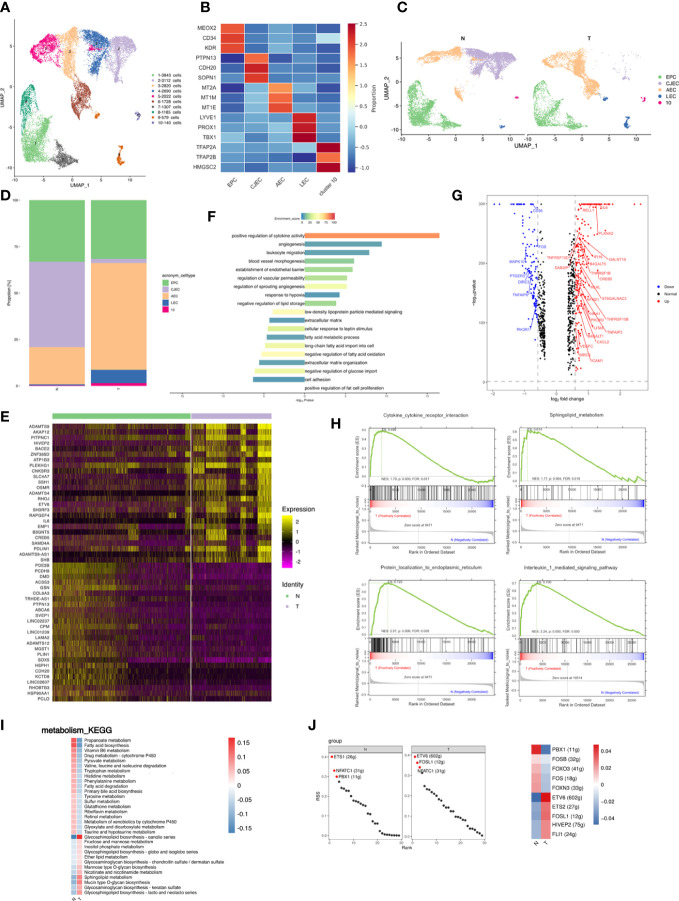
Breast cancer promotes the inflammatory profile of endothelial cells in adipose tissues. **(A)** UMAP plot of 10 clusters of endothelial cells from the 6 samples profiled in this study, with each cell color coded to indicate the associated subclusters and cell numbers. **(B)** Heatmap showing the expression of endothelial subpopulation marker genes in each cluster. The color changes from blue to red, indicating a low to high fold change. **(C)** UMAP plot of all endothelial subpopulations annotated in each group, with each cell color coded to indicate the associated subclusters. **(D)** The relative ratio of endothelial cells in N and T group shown using bar plots, with each cell color coded to indicate the associated subclusters. **(E)** Heatmap showing the top 25 differential expression genes (up- or down-regulated). The color changes from blue to red, indicating a low to high fold change. **(F)** Barplot showing the significance levels of the top 10 most significantly enriched pathways in all DEGs. The left side of the coordinate axis represents the enriched functional pathway of down-regulated genes; The right side of the coordinate axis represents the enriched functional pathway of up-regulated genes. **(G)** Expression of genes (N versus T) involving in inflammatory (GO:0006954) in endothelial cells. **(H)** The top 4 GSEA analysis results of angiogenesis associated endothelial subpopulation. **(I)** Heatmap indicating the enrichment of metabolism signaling pathway on all endothelial cells in N and T group. **(J)** The regulon specificity score (RSS) ranking plot of top 3 TFs in all endothelial cells in N and T group. The abscissa represents the ranking, and the ordinate represents the RSS score. The regulator with higher RSS may be related to the specificity of this cell group (left). Heatmap showing the regulon activity in N and T group. Rows represent different regulons, and columns represent different cell populations. The color changes from blue to red, indicating a low to high RSS specificity score. The higher the RSS score, the stronger the specificity of regulon in this cell group (right). The number of target genes shown in brackets.

Endothelial cells accounted for a large proportion of cell types showing cancer-related changes and were separated into 10 distinct subpopulations (**Figure 3A**; **Supplementary Table S4**). Subpopulations were annotated according to marker genes and pathway analysis as follows: cluster 1, 7 and 8 were endothelial progenitor cells (EPCs; expressing e.g., MEOX2, CD34 and KDR); cluster 2 and 4 expressed genes, such as PTPN13, CDH20, and SPON1, related to cell junction and adhesion (cell junction-associated endothelial cells; CJECs) with implications for cell migration, invasion and epithelial-mesenchymal transition (EMT); cluster 3, 5 and 6 expressed metallothionein family member genes, such as MT2A, MT1M and MT1E, involved in angiogenesis (angiogenesis-associated endothelial cells; AECs); cluster 9 comprised lymphatic endothelial cells (LECs; expressing e.g., TBX1, PROX1 and LYVE1); cluster 10 expressed genes associated with endothelial cell development and homeostatic maintenance (expressing e.g., TFAP2A, TFAP2B, and HMGCS2) (**Figure 3B**; **Supplementary Figure S4A**). Compositional analysis revealed increases in percentages of clusters 3, 5, 6 and 9, and decreases in clusters 2 and 4 in T tissues (**Figures 3C, D**; **Supplementary Figures S4B, C**). Analysis of DEGs identified 246 upregulated and 138 downregulated genes in T, the top 25 of which are presented as a heatmap (**Figure 3E**). Analysis of DEG function showed that upregulated genes were enriched for angiogenesis, vascular homeostasis, and inflammation. Downregulated genes were enriched for cell adhesion, extracellular matrix, and metabolism (**Figure 3F**). Overall indications were that breast cancer promoted angiogenesis related processes but damaged cell junction and adhesion, contributing to migration and invasion of cancer cells.

Endothelial cells also produce cytokines. Analysis of genes relating to cytokine release by endothelial cells showed that the top 3 upregulated cytokines in the T group were IL-6, RELLE and PLXAN2, suggesting that breast cancer promoted an inflammatory state among endothelial subpopulations (**Figure 3G**). IL-6 is an inflammatory factor and a multifunctional cytokine with extensive functions. RELL1 is a member of the tumor necrosis factor (TNF) receptor family and belongs to the receptor expressed in the lymphoid tissues (RELT) family. RELL1 activates the pro-inflammatory pathway by binding to TNF receptor-related factor 1 (TRAF1) (46).

Relative proportions of AECs were altered in breast cancer and increased to become the most abundant subpopulation of T. GSEA of AECs showed enrichment of cytokine-receptor interactions, sphingolipid metabolism, protein localization to endoplasmic reticulum (ER), and IL-1 signaling pathway in AECs (**Figure 3H**), indicating stimulation of the inflammatory response and angiogenesis (47–49).

Glycosphingolipid biosynthesis and sphingolipid metabolism was enriched in the T group (**Figure 3I**), indicating that breast cancer promoted an inflammatory state among endothelial subpopulations. SCENIC data showed enrichment of ETS1 regulons in N tissues and of ETV6 regulons in T tissues (**Figure 3J** left). The T group thus presented a high activity of inflammatory regulons, similar to the situation in macrophages (**Figure 3J** right).

In conclusion, breast cancer promoted an inflammatory state of endothelial subpopulations and angiogenesis, decreasing subclusters related to cell junctions, which may contribute to the inflammatory state and metastasis in breast cancer.

### 3.4 Breast cancer induced a decrease in the preadipocyte subpopulation

**Figure 4:**
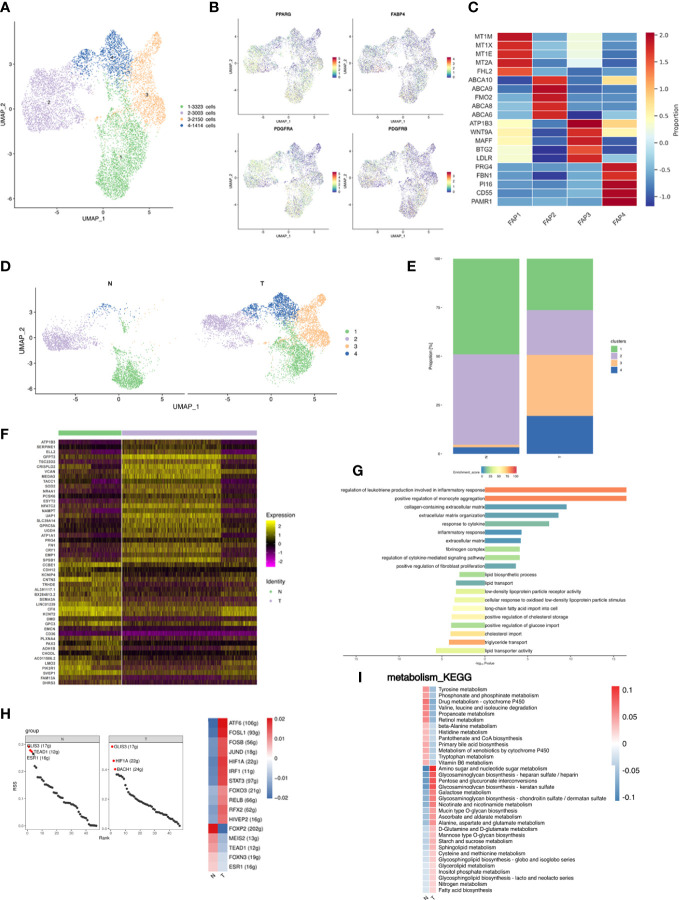
Fibro-adipogenic progenitors are shifted toward less adipogenic phenotype in breast cancer. **(A)** UMAP plot of 4 clusters of FAPs from the 6 samples profiled in this study, with each cell color coded to indicate the associated subclusters and cell numbers. **(B)** UMAP plot of preadipocyte marker genes PPARG, FABP4, PDGFRA and PDGFRB. **(C)** Heatmap showing the expression of FAP subpopulation marker genes in each cluster. The color changes from blue to red, indicating a low to high fold change. **(D)** UMAP plot of all FAP subpopulations annotated in each group with each cell color coded to indicate the associated subclusters. **(E)** The relative ratio of FAPs in N and T group shown using bar plots, with each cell color coded to indicate the associated subclusters. **(F)** Heatmap showing the top 25 differential expression analysis of genes (up- or down-regulated). The color changes from blue to red, indicating a low to high fold change. **(G)** Barplot showing the significance levels of the top 10 most significantly enriched pathways in all DEGs. The left side of the coordinate axis represents the enriched functional pathway of down-regulated genes; The right side of the coordinate axis represents the enriched functional pathway of up-regulated genes. **(H)** The regulon specificity score (RSS) ranking plot of top 3 TFs in N and T group. The abscissa represents the ranking, and the ordinate represents the RSS score. The regulator with higher RSS may be related to the specificity of this cell group (upper). Heatmap showing the regulon activity in N and T group. Rows represent different regulons, and columns represent different cell populations. The color changes from blue to red, indicating a low to high RSS specificity score. The higher the RSS score, the stronger the specificity of regulon in this cell group (lower). The number of target genes shown in brackets. **(I)** Heatmap indicating the enrichment of metabolism signaling pathway on all FAPs in N and T group.

FAPs consist of fibroblasts, preadipocytes, and stem cells which were re-clustered into 4 distinct subpopulations (**Figure 4A**). FAP1 and FAP2 expressed preadipocyte marker genes PDGFRA and PPARG (**Figure 4B**). Preadipocyte differentiation involves a sequence including cell cycle arrest, expansion of mitotic clones, post-mitotic growth arrest and terminal differentiation (50) and cell cycle- and cell apoptosis-related genes are fundamental to cell proliferation and growth. FAP1 were proliferative preadipocytes characterized by high expression of genes modulating cell proliferation (expressing e.g., FHL2, MT1M, MT1X, MT1E, and MT2A); FAP2 were enriched for immune- and inflammation-related functions and expressed ABCA transporters involved in cholesterol uptake and efflux (expressing e.g., ABCA10, ABCA8, ABCA6, and ABCA9); FAP3 were enriched for cell cycle-related functions and expressed similar marker genes to FAP1; FAP4 were similar to previously reported fibro-inflammatory progenitors (FIPs) with high expression of fibro-genic genes (expressing e.g., PRG4, FBN1, CD55, PI16, and PAMR1). FIPs lack adipogenic capacity, exert pro-fibrogenic/pro-inflammatory phenotype, and display an anti-adipogenic effect (51) (**Figure 4C**; **Supplementary Table S5**). Thus, breast cancer may promote a less adipogenic phenotype in FAPs (**Figures 4D, E**). three patients were independently analyzed to investigate whether differences in cluster proportions in N and T were significant. Cluster 4 (FIPs) showed a significant increase in T (p=0.02) (**Supplementary Figures S5A, B**).

Analysis of DEGs identified 240 upregulated and 67 downregulated genes in T, the top 25 of which are presented as a heatmap (**Figure 4F**). Analysis of DEG function showed that upregulated genes were involved in extracellular matrix organization and inflammatory pathways. Downregulated genes were enriched for involvement in lipid transport (**Figure 4G**). Comparison of FAP gene expression between the N and T groups identified unique functions enriched in DEGs, indicating differences in FAPs between normal breast and cancer associated adipose, and showed 307 GEGs of which top 25 are shown in a heatmap (**Figure 4F**). Stimulation of the profibrotic phenotype in fibroblasts and preadipocytes by the macrophage-induced inflammatory response has been previously reported and is consistent with our data (52).

The analysis of TFs showed that both N and T were co-enriched for the GLIS3 regulon. The N group was additionally enriched in TEAD1, ESR1, FOXP2 and MEIS2 regulons, and the T group in HIF1A, BACH1, ATF6 and FOSL1 regulons (**Figure 4H**). Metabolic alterations are shown in **Figure 4I**. The T group was enriched in amino sugar and nucleotide metabolism and glycosaminoglycan metabolism pathways.

In conclusion, 4 distinct FAP subpopulations were identified, and breast cancer decreased the proportion of preadipocytes and upregulated genes involved in the profibrotic phenotype and inflammatory response.

### 3.5 Breast adipose tissue consisted of distinct subpopulations of adipocytes

**Figure 5:**
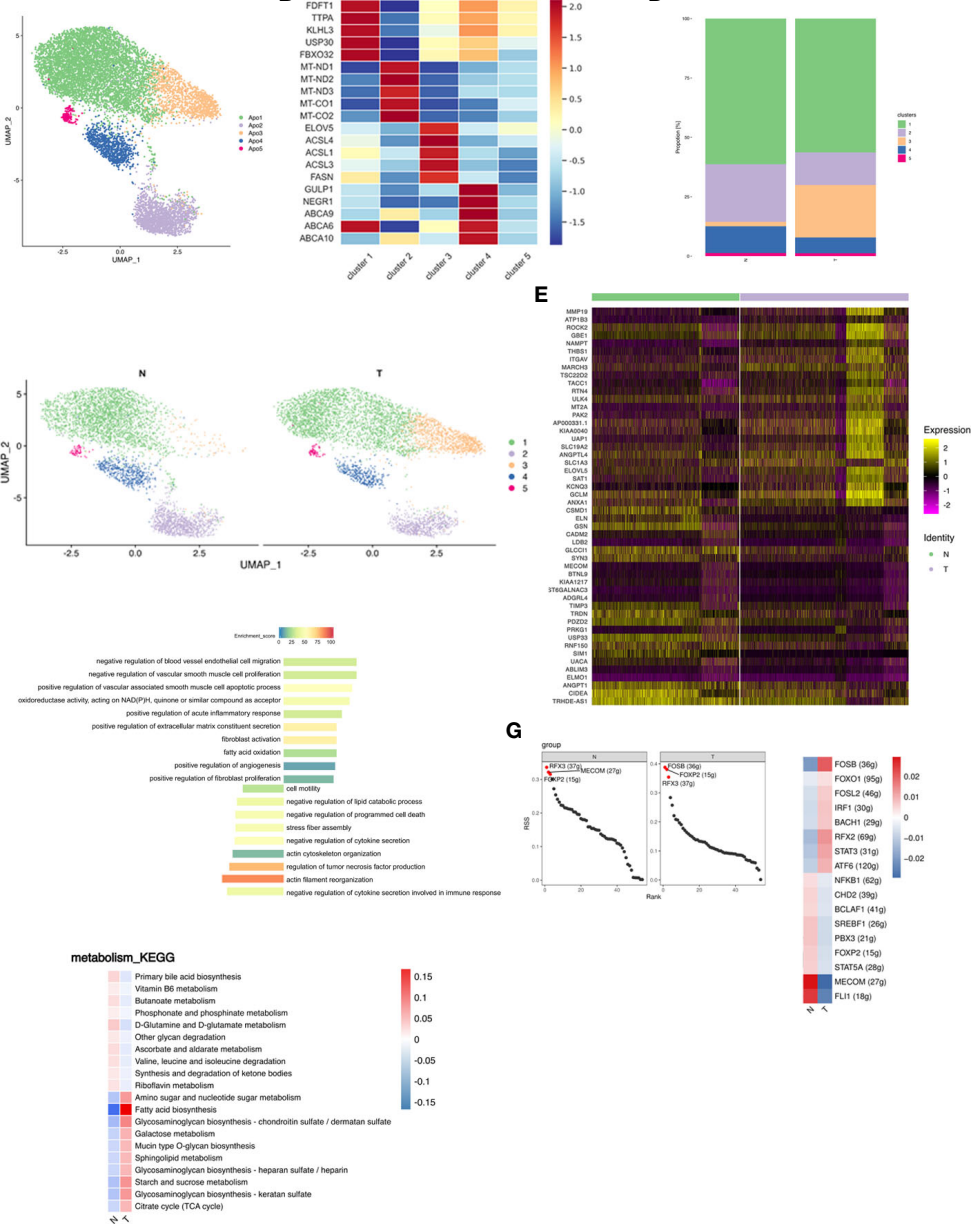
Adipocyte subpopulations in adipose tissue are changed by breast cancer. **(A)** UMAP plot of 5 clusters of adipocytes from the 6 samples profiled in this study, with each cell color coded to indicate the associated subclusters and cell numbers. **(B)** Heatmap showing the expression of adipocyte subpopulation marker genes in each cluster. The color changes from blue to red, indicating a low to high fold change. **(C)** UMAP plot of all adipocyte subpopulations annotated in each group with each cell color coded to indicate the associated subclusters. **(D)** The relative ratio of adipocytes in N and T group shown using bar plots, with each cell color coded to indicate the associated subclusters. **(E)** Heatmap showing the top 25 differential expression analysis of genes (up- or down-regulated). The color changes from blue to red, indicating a low to high fold change. **(F)** Barplot showing the significance levels of the top 10 most significantly enriched pathways in all DEGs. The left side of the coordinate axis represents the enriched functional pathway of down-regulated genes; The right side of the coordinate axis represents the enriched functional pathway of up-regulated genes. **(G)** The regulon specificity score (RSS) ranking plot of top 3 TFs in N and T group. The abscissa represents the ranking, and the ordinate represents the RSS score. The regulator with higher RSS may be related to the specificity of this cell group (left). Heatmap showing the regulon activity in N and T group. Rows represent different regulons, and columns represent different cell populations. The color changes from blue to red, indicating a low to high RSS specificity score. The higher the RSS score, the stronger the specificity of regulon in this cell group (right). The number of target genes shown in brackets. **(H)** Heatmap indicating the enrichment of metabolism signaling pathway on all adipocytes in N and T group.

Adipocytes were re-clustered and separated into 5 distinct subpopulations (**Figure 5A**). Cluster 1 highly expressed cholesterol synthesis related genes (e.g., FDFT1, TTPA, KLHL31, USP30, and FBXO32); cluster 2 expressed oxidative phosphorylation (OXPHOS) -related genes (e.g., MT-ND1, MT-ND2, MT-ND3, MT-CO1, and MT-CO2), indicating that this subpopulation may remove excess metabolites from the circulatory system (53); cluster 3 expressed genes involved in lipid biosynthesis (e.g., ELOVL5, ACSL4, ACSL1, ACSL3, and FASN); cluster 4 expressed genes associated with cholesterol efflux and lipid transport (e.g., GULP1, NEGR1, ABCA9, ABCA6, and ABCA10), suggesting that the primary source of lipids for this subcluster may be uptake rather than *de novo* lipogenesis; cluster 5 was not further analyzed due to low cell numbers (**Figure 5B**; **Supplementary Table S6**). Proportions of the 5 clusters in each patient were shown in **Supplementary Figure S6A**. Differences in cluster proportion in N and T were analyzed for significance in three patients (**Supplementary Figure S6B**). Breast cancer shifted the ratio of adipocytes from a lipolytic phenotype towards a lipogenic and inflammatory phenotype (**Figures 5C, D**). Elevated FAs production induces adipocyte inflammation and maintains glucose uptake *via* feedback, whereas fatty acid oxidation prevents FA induced inflammation, oxidative stress, and insulin resistance (54). Inhibition of fatty acid oxidation promotes cytokine release and inflammation. Comparison of gene expression in adipocytes between the N and T groups showed 116 upregulated and 30 downregulated genes in T, the top 25 of which are shown in a heatmap (**Figure 5E**). The upregulated DEGs were associated with endothelial cell function, angiogenesis, fibroblastic response, acute inflammatory response and extracellular matrix, and the downregulated DEGs were enriched in cytoskeletal organization, cell motility and stress fiber assembly (**Figure 5F**), suggesting that breast cancer led to derangement in cellular metabolism.

The N group showed enrichment of the MECOM and the T group of the FOSB regulons (**Figure 5G** left). MECOM and FLI1 activities were decreased, while breast cancer promoted FOSB and RFX2 activities (**Figure 5G** right). The most significantly altered metabolic pathway was fatty acid biosynthesis (**Figure 5H**).

In summary, breast cancer decreased lipid uptake and the lipolytic phenotype and caused a switch to lipid biosynthesis *via* deranged cellular metabolism adipocytes.

### 3.6 Mapping the developmental track of adipocytes by pseudotime state transition

**Figure 6:**
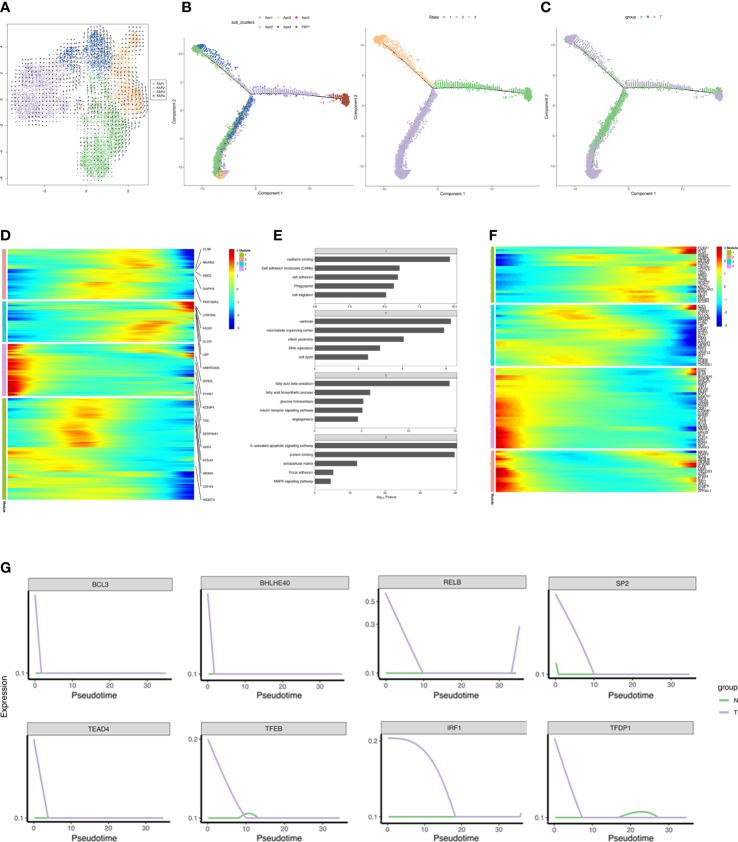
Reconstruction of adipogenesis *in vivo.* **(A)** RNA velocity analysis of FAPs. The velocities were layered on top of the FAP subpopulations in UMAP space (**Figure 4A**). The results of RNA velocity are mapped to the UMAP dimensionality reduction cluster diagram. The points in the diagram represent cells, the colors represent different cell types/cell clusters, and the arrow direction represents the cell differentiation direction predicted by the algorithm. **(B, C)** Monocle analyses showing the development of FAP and adipocyte along the pseudotime trajectory. The left figure in B shows the pseudo-time trajectory of cell cluster differentiation, and each color represents a cell cluster, the right figure in B shows the pseudo-time trajectory of cell differentiation state, and each color represents a cell state. **(C)** shows the pseudo-time trajectory of cell differentiation in different group, and each color represents a group. **(D)** Heatmap showing the differentially expressed genes in each module (along pseudo-time across the trajectory) in T group, and we listed the top 5 DEGs in each module in the figure. Each row represents a gene, the horizontal axis from left to right represents the time from morning to night, and the color from blue to red represents the expression of genes from low to high. Clustering of genes with similar expression patterns in the time development track. The colors represent different module. **(E)** Barplot showing the significance levels of the top 5 most significantly enriched pathways in each gene group (all DEGs in **D**). The numbers in the gray grid above each histogram represented the corresponding module in panel **(D)**. **(F)** Heatmap showing expressions of transcriptional regulators differentially expressed along pseudo-time across the trajectory. Each row represents a gene, the horizontal axis from left to right represents the time from morning to night, and the color from blue to red represents the expression of genes from low to high. Clustering of genes with similar expression patterns in the time development track. **(G)** Pseudotime ordered single-cell expression trajectories of TFs in N and T group. The horizontal axis from left to right represents the time from early differentiation to late differentiation. The vertical axis represents the amount of gene expression. Lines with different colors represent different groups.

SnRNA-seq analysis allowed simultaneous profiling of FAPs and mature adipocytes and a pseudotime trajectory was applied to detect the differentiation trajectory of adipogenesis *in vivo*. FAP1 were found to be *bona fide* preadipocytes and expressed the PDGFRB^+^ characteristic of adipocyte progenitors (55–57) (**Figure 4B**). However, RNA velocity analysis (58) did not detect a definite transition among the 4 FAP subclusters (**Figure 6A**). A continuum of cell states with two distinct trajectories from state 1 through to states 2 and 3, common origin with divergent fates, were shown by mapping the trajectory connecting FAPs and adipocytes. State 1 represented adipogenesis and states 2 and 3 specialization of mature adipocytes into distinct subclusters (**Figure 6B**). N and T group trajectories overlapped (**Figure 6C**). Each patient’s differentiation status was shown in **Supplementary Figure S7**.

Differential gene expression across the trajectory was analyzed in the T group which was divided into 4 groups based on temporal expression patterns (**Figure 6D**). ECM, focal adhesion, and MAPK signaling pathway associated genes were expressed in early differentiation, while genes specific to fatty acid metabolism, angiogenesis, glucose homeostasis and insulin receptor signaling were detected in late differentiation. Genes involved in cell adhesion, migration, and cell cycle were abundantly expressed during differentiation (**Figure 6E**). DEGs included 98 transcriptional regulators, indicating the complexity of the transcriptional reprogramming related to adipogenesis. TFs analysis indicated progression from stemness-related TFs to adipogenic TFs. Adipogenic transcriptional waves were detected, such as repression of KLF2, KLF7, ATF3, RORA, and EGR1 and induction of KLF6, ARID5B, E2F3, and PPARG, as has been previously reported (59–63) (**Figure 6F**). Strikingly, breast cancer induced several anti-adipogenic TFs at the beginning of differentiation which declined with time, indicating that breast cancer repressed adipogenesis (**Figure 6G**). Overall, the reconstructed adipogenesis trajectory illuminates the influences of breast cancer on the adipogenesis differentiation.

### 3.7 Complex intercellular communication networks in adipose tissues

**Figure 7:**
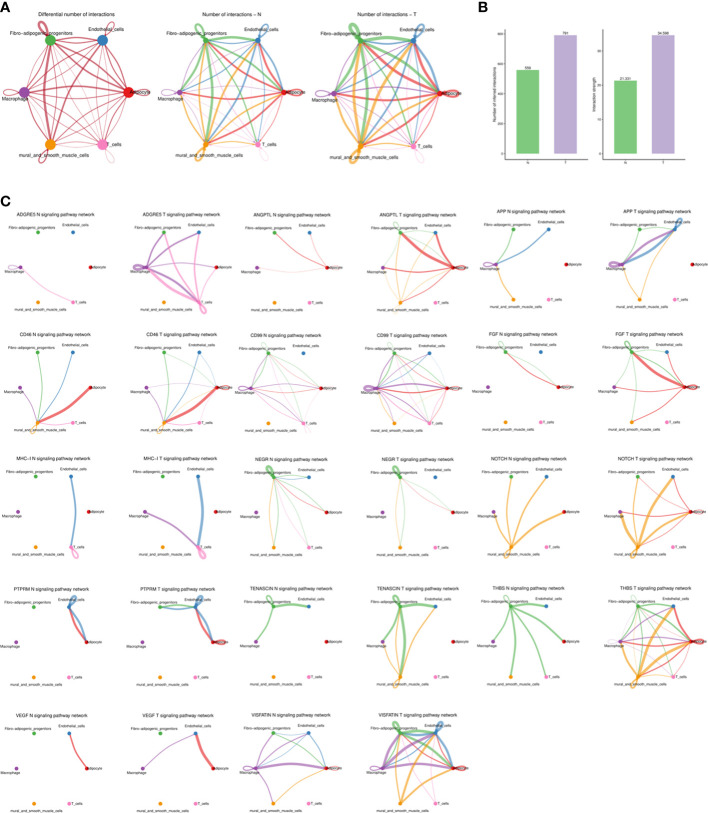
The dense network and multiple cellular communications. **(A)** Circos plot showing the interactions between ligands and receptors across cell types in two groups (left), N group (middle), and T group (right). Different colors in the circle plot represent different cell groups. The thicker the line is, the more interactions between cell types are detected. The color of the line is the same as that of the ligand cell, that is, the cell that sends the signal. **(B)** Barplot showing the number and strength of interactions across cell types in two groups. The left figure is the statistical histogram of the number of intercellular interactions, and the right figure is the statistical histogram of the intensity of intercellular interactions. The abscissa represents different groups. **(C)** The association of different signaling pathways among different cell types of each group. Different colors in the circle plot represent different cell groups. The figure can view the number of interactions between cell types under a certain signal pathway. The thicker the line is, the more interactions between cell types are detected. The color of the line is the same as that of the ligand cell, that is, the cell that sends the signal.

In order to systematically assess the complex cellular response, ligand-receptor communication networks were mapped to illuminate the cellular behavior and interaction with neighbouring cells. Intercellular interactions were predicted according to specific protein complexes and a network among all cell types in the N and T groups generated (**Figure 7A**). Breast cancer upregulated both the number and strength of interactions (**Figure 7B**).

Higher levels of several ligand-receptor communications, such as THBS1-CD47, THBS1-CD36, SEMA3C-NRP2/PLXNA2, PROS1-AXL, NAMPT-ITGA5/ITGB1, LAMC1-CD44, LAMA4-CD44, LAMA3-CD44, FN1-CD44, FN1-ITGAV/ITGB1, COL4A1-ITGA11/ITGB1, ANGPTL4-SDC2, and ADGRE5-CD55 were found in breast cancer (**Supplementary Figure S8**) and communications related to angiogenesis, inflammation and immune regulation were promoted (**Figure 7C**).

## 4 Discussion

Breast cancer is the most common cancer among females and shows rapidly increasing incidence. Despite progress in pathogenesis and treatment which has decreased mortality rate, breast cancer remains the second leading cause of cancer-related deaths in women (64). The breast comprises fibroglandular and adipose tissues, the latter covering the majority of the breast and modulating the interaction of all components of the breast microenvironment. Breast cancer adipose stores energy and secretes factors necessary for tumor cell survival. Therefore, breast adipose tissue is considered a master modulator of breast cell physiology (65).

SnRNA-seq was used in the current work to characterize normal and cancer-adjacent adipose transcriptomes from postmenopausal breast cancer patients at single-nucleus resolution to define cell heterogeneity and cell-type composition. A comprehensive cellular atlas of breast adipose tissues was identified, *in vivo* developmental trajectories defined and subpopulation plasticity ascertained in response to breast cancer. Interaction networks were also explored. ScRNA-seq data revealed altered cell types and localized expression of pathogenic genes in distinct cell subclusters. Normal and cancer-adjacent adipose tissues were generally similar but some differences in cell subtypes, reflected by gene expression and cell composition, were found.

T cells, especially the CD8^+^ subset, are involved in adipose tissue inflammation. These cells are activated by obese adipose tissues and recruited to activate adipose-resident macrophages (66) which infiltrate obesity-related adipose tissue resulting in the inflammation and insulin resistance. Vijay et al. identified a dysfunctional adipose-resident T cell subcluster which expressed metallothionein genes with relevance to adipose inflammation and potential insulin resistance (29). Han et al. found that activated CD8+ T cells contributed specifically to increased adipose tissue IFNG expression in cachexia patients and had a pro-catabolic effect on adipocytes *in vitro*. Such observations may indicate mechanisms underlying the chronic inflammation and adipose wasting in cancer-associated cachexia (CAC) (67). Macrophages constituted the majority of immune cells in the current study and the general components of T cells did not vary greatly between N and T samples. T cells could not be divided into different subclusters and detailed analysis was not performed.

Macrophages have microenvironment-specific phenotypes to maintain tissue development and internal environment stability. Disorders of ATMs precipitate many pathologies including inflammatory diseases, fibrosis and cancer and ATMs are potential therapeutic targets. TAMs support tumor progression by blocking anti-tumor immunity and secreting pro-angiogenesis and EMT reactivation factors, thereby enhancing metastasis (68). Macrophage plasticity and stress-specific responses result in heterogeneous macrophage subpopulations with complex phenotypes (69). Difficulties in clarifying specific functions of heterogeneous macrophage subpopulations may impede development of disease therapies. The current macrophage analysis identified the increase in the MARCO^+^PLAUR^+^ subcluster. MARCO expression is considered to define a M2-like macrophage subtype with an immunosuppressive gene signature which is expressed by tumor-promoting macrophages producing a worse prognosis (12, 13, 23). PLAUR may be involved in tumor immunity and leads to tumor invasion and metastasis by regulating extracellular matrix degradation and tissue remodeling (15, 16, 25–27). Macrophage subpopulations with high expression of the lipid transporter, CD36, were also found. Lipid metabolism is central to TAM differentiation and function and a tumor inhibitory effect of CD36 knockdown has been shown. CD36 has also been associated with TME-induced tumor growth and metastasis and inhibition of the immune response (70, 71). ScRNA-seq data of the current study indicated increased MARCO^+^ and CD36^+^ macrophage subpopulations with immunosuppressive functions in breast cancer.

Endothelial cells are involved in tumor cell infiltration into adjacent tissues, migration through endothelial cells (intravasation), survival in the bloodstream, extravasation and colonization of the target organ. Non-activated, quiescent endothelial cells exhibit an anti-inflammatory and anti-coagulant phenotype. Endothelial cells stimulated to proliferate and migrate by tumor cells, participate in angiogenesis. Tumor-derived growth or chemotactic factors recruit and activate monocytes to differentiate into TAMs which secrete cytokines and growth factors to promote angiogenesis (72). Changes in endothelial subpopulations associated with cell junctions, angiogenesis and inflammation indicated that breast cancer caused endothelial dysfunction and angiogenesis, contributing to tumor invasion and metastasis.

Adipocytes account for less than 20% of the total cells in adipose tissue but occupy the largest mass and volume. The remaining 80% of adipose tissue cells are stromal vascular cells (SVCs), including immune cells, endothelial cells, adipocyte stem cells and fibroblasts (73). Coordination of adipocytes and SVCs is essential for homeostasis and systemic metabolism of adipose tissue. In some endocrine diseases, benign and malignant tumors cause hormonal disorders and persistent inflammation, impacting the ECM, promoting endoplasmic reticulum stress and adipose tissue inflammation to cause adipose tissue disorders (74). Breast cancer led to a major shift from OXPHOS to lipogenesis in adipocytes and induced the inflammatory phenotype in adipocytes of the present study. Distinct adipocyte subclusters are likely to represent different phenotypic states due to adipocyte plasticity, consistent with the current trajectory analysis. Adipogenesis was observed to favor OXPHOS-type adipocytes. We suggest that the reduction of OXPHOS associated adipocyte subpopulations in breast cancer adjacent adipose tissues is a consequence of adipocyte plasticity rather than elimination by necrosis or apoptosis.

FAPs were clustered into 4 distinct subpopulations, where FAP3 and FAP4 represented fibroblasts that did conform to the adipogenic lineage, according to velocity analysis. However, RNA velocity analysis indicated that transcriptional programs may favor adipogenesis, driving some preadipocytes toward fibroblasts (**Figure 6A**). Hence, FAP3 or FAP4, which increased in cancer-adjacent adipose tissues, may influence fibrotic outcomes in adipose tissue in breast and pancreatic cancer, consistent with previous reports (75).

Increased ligand-receptor communications associated with inflammation, fibrosis, and angiogenesis in cancer-adjacent adipose tissues were detected in the current work, indicating an imbalanced immune environment in breast cancer.

The enrichment of TFs and metabolism-based KEGG analysis showed that breast cancer induced an inflammatory phenotype by upregulating the enrichment of pro-inflammatory TFs and promoting metabolic pathways related to inflammation, such as glycosphingolipid, lipid biosynthesis and glycosaminoglycan metabolism.

We acknowledge that a limitation of our study is that there are only three samples which could have impacted some results and increased heterogeneity, and further studies are needed to distinguish breast cancer types and perform experimental validation of heterogeneity and diversity and its impact on occurrence and development of breast cancer. In our data, some clusters are patient dependent and also some adipocyte differentiation state, such as clusters 2 and 3 in macrophages, and in **Figure 6B**, state 3 is patient 53 specific, so we removed the data of patient 53 and obtained the similar results that we observed the marked increase in the proportions of both MARCO^+^PLAUR^+^, LAMs, AECs, LECs and FIPs (data were unshown), indicating the enhanced pro-tumor functions and inflammatory phenotype in breast cancer adjacent adipose tissues. While, it is necessary to collect more patient samples including different breast cancer types to validate our conclusions. In a mouse study, scRNA-seq was used to study adipocyte de-differentiation in the tumor-microenvironment (8). However, the tumor impact in adipocytes remains to be investigated in breast cancer patients. The current study gives some insights into breast cancer associated adipose tissues.

## 5 Conclusions

In conclusion, a comprehensive atlas of breast adipose tissue plasticity at single-nucleus resolution in response to breast cancer was generated. Considerable alternations in gene expression and cellular composition were found. The adipogenic trajectory *in vivo* was constructed and crosstalk between distinct cell types inferred. The current scRNA-seq data provides a solid foundation for further hypothesis-driven investigation of breast cancer pathogenesis, leading to more effective treatment strategies.

## Data availability statement

The datasets presented in this study can be found in online repositories. The names of the repository/repositories and accession number(s) can be found below: NCBI via accession ID: PRJNA932038.

## Ethics statement

The studies involving human participants were reviewed and approved by Zhengzhou Center Hospital. The patients/participants provided their written informed consent to participate in this study.

## Author contributions

LNT conceived the project and designed the study. LNT with the help from TTL and JX. JX and YPH provided breast cancer adipose tissues. LNT and TTL analyzed data and wrote the manuscript. All authors read and approved the final manuscript. All authors contributed to th article and approved the submitted version.
