# Corrigendum: Diversity and heterogeneity in human breast cancer adipose tissue revealed at single-nucleus resolution

**DOI:** 10.3389/fimmu.2023.1213786

**Published:** 2023-05-31

**Authors:** Lina Tang, Tingting Li, Jing Xie, Yanping Huo, Jianping Ye

**Affiliations:** ^1^ Metabolic Disease Research Center, Zhengzhou Central Hospital Affiliated to Zhengzhou University, Zhengzhou, China; ^2^ Department of Cell Biology, Key Laboratory of Cell Biology, National Health Commission of the PRC and Key Laboratory of Medical Cell Biology, Ministry of Education of the PRC, China Medical University, Shenyang, Liaoning, China; ^3^ Department of Breast Surgery, Zhengzhou Central Hospital Affiliated to Zhengzhou University, Zhengzhou, China; ^4^ Center for Advanced Medicine, College of Medicine, Zhengzhou University, Zhengzhou, China; ^5^ Research Center of Basic Medicine, Academy of Medical Sciences, Zhengzhou University, Zhengzhou, Henan, China

**Keywords:** breast cancer, adipose tissue, single-cell RNA sequencing, diversity, heterogeneity

In the published article, there was an error in affiliation 1. Instead of “Advanced Medical Research Center of Zhengzhou University, Zhengzhou Central Hospital Affiliated to Zhengzhou University, Zhengzhou, China”, it should be “Metabolic Disease Research Center, Zhengzhou Central Hospital Affiliated to Zhengzhou University, Zhengzhou, China”.

In the published article, there was an error in the author list, and author Jianping Ye was erroneously excluded. The corrected author list appears below.

Lina Tang^1^, Tingting Li^2^, Jing Xie^3^, Yanping Huo^3^*, Jianping Ye^1,4,5^*

The authors apologize for these errors and state that this does not change the scientific conclusions of the article in any way. The original article has been updated.

