# Supplementary material for: Diversity and heterogeneity in human breast cancer adipose tissue revealed at single-nucleus resolution
Source: Front Immunol. 2023 Apr 21;14:1158027. doi: 10.3389/fimmu.2023.1158027 (PMC10160491; doi:10.3389/fimmu.2023.1158027)

Supplementary Fig. S1. Study flow chart


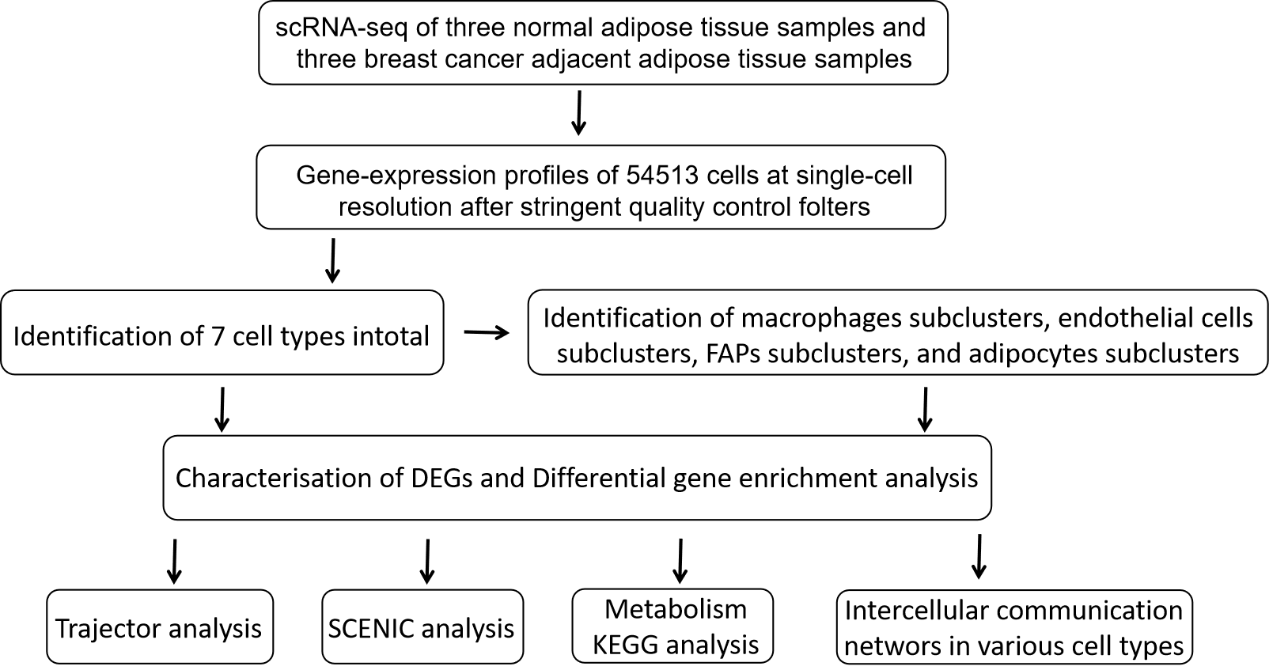


Supplementary Fig. S2. snRNA-seq of breast cancer adipose tissues

1. Proportions of 21 clusters in each group
2. UMAP plot of 21 clusters in each group
3. Proportion of all 21 clusters in each patient
4. Proportion of all cell types in each patient


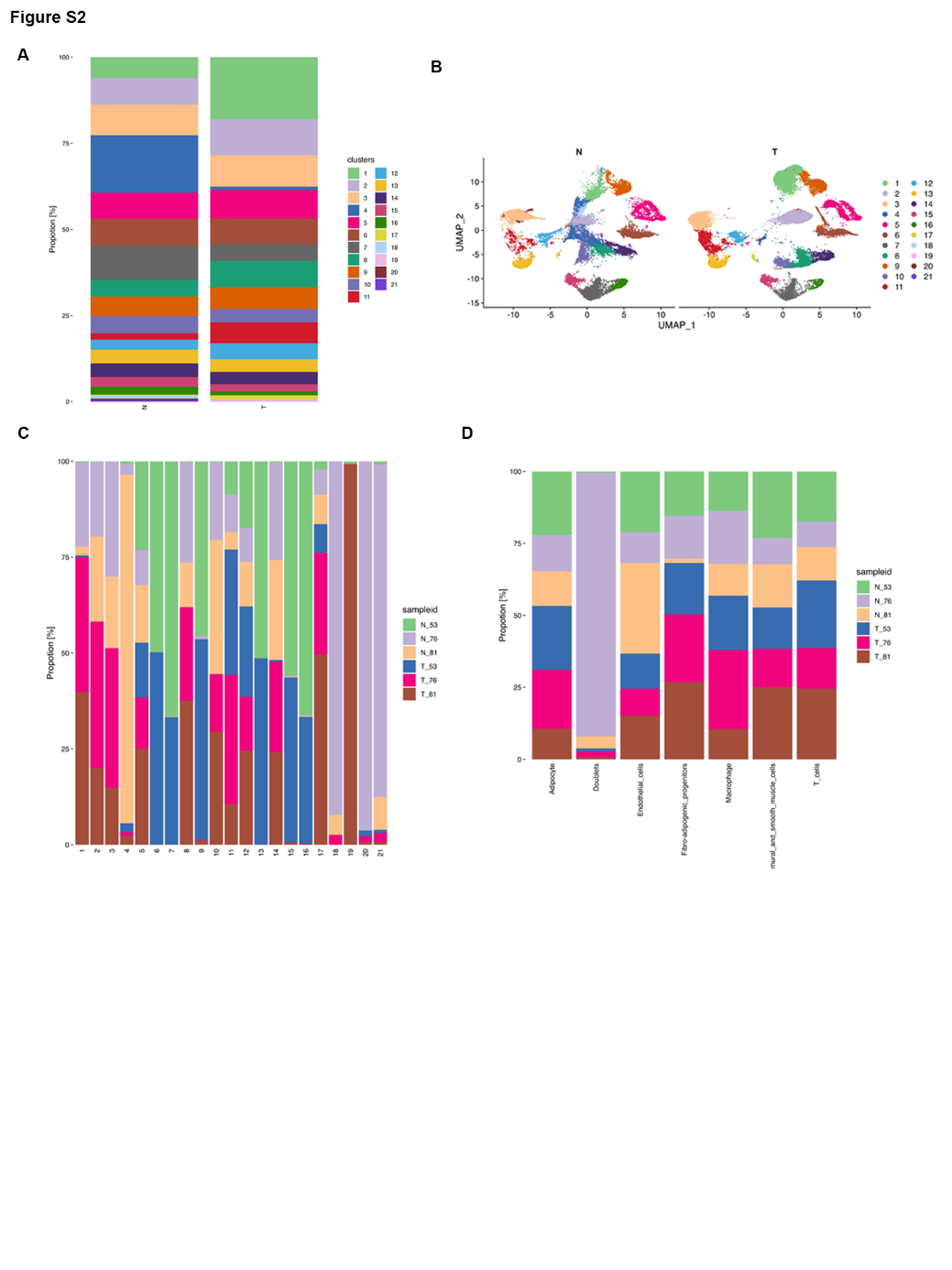


Supplementary Fig. S3. Proportion of 8 macrophage clusters in each patient

1. Violin plot of LYVE1 and SELENOP for each cluster
2. The proportion of 8 macrophage clusters in each patient
3. The average fraction (relative to the total number of macrophage nuclei) of each subpopulation in N and T group


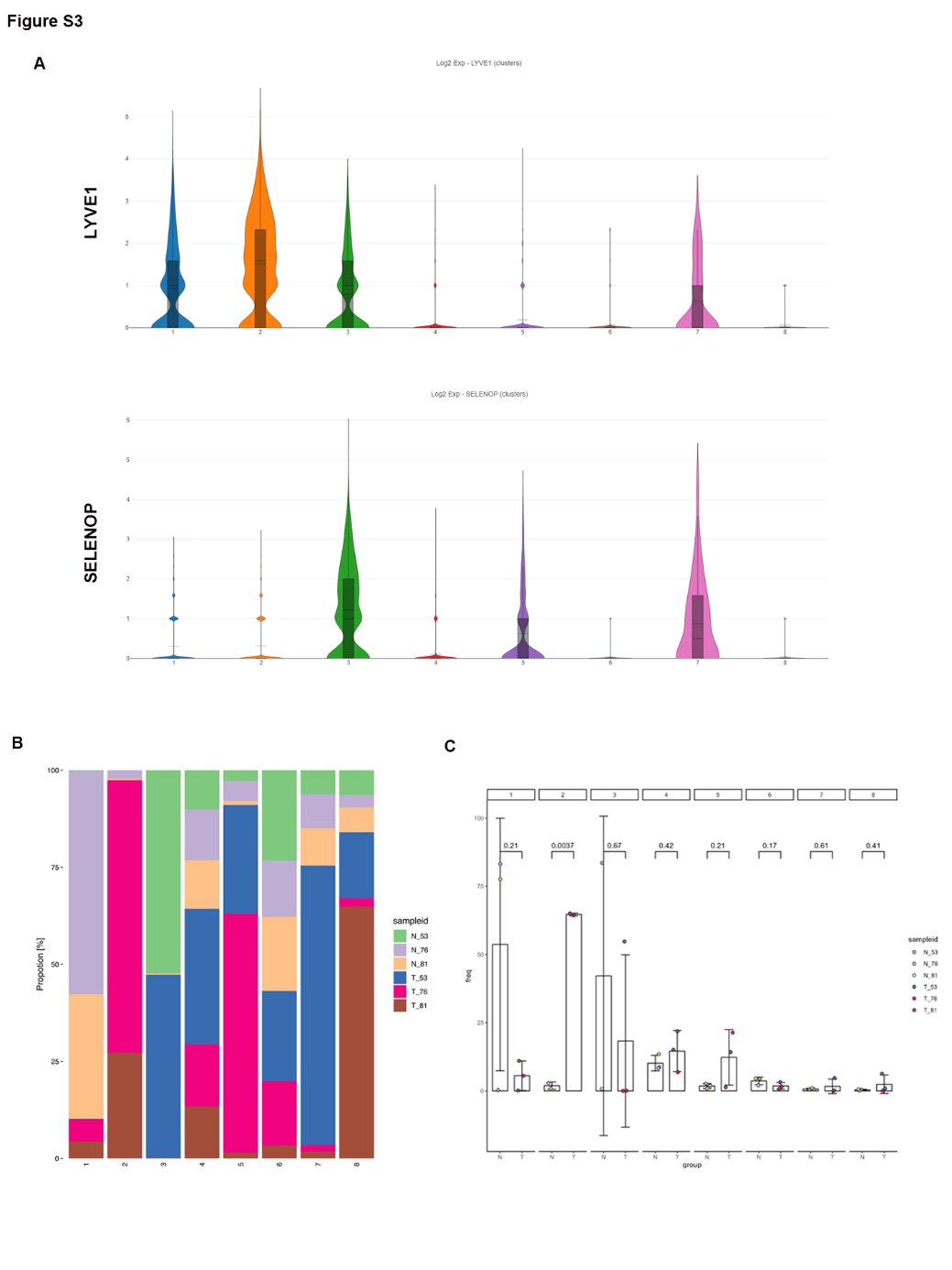


Supplementary Fig. S4. Influences of Breast Cancer on Endothelial Cells

1. Violin plot of marker genes for each cluster
2. Proportion of 10 endothelial clusters in each patient
3. The average fraction (relative to the total number of endothelial nuclei) of each subpopulation in N and T group


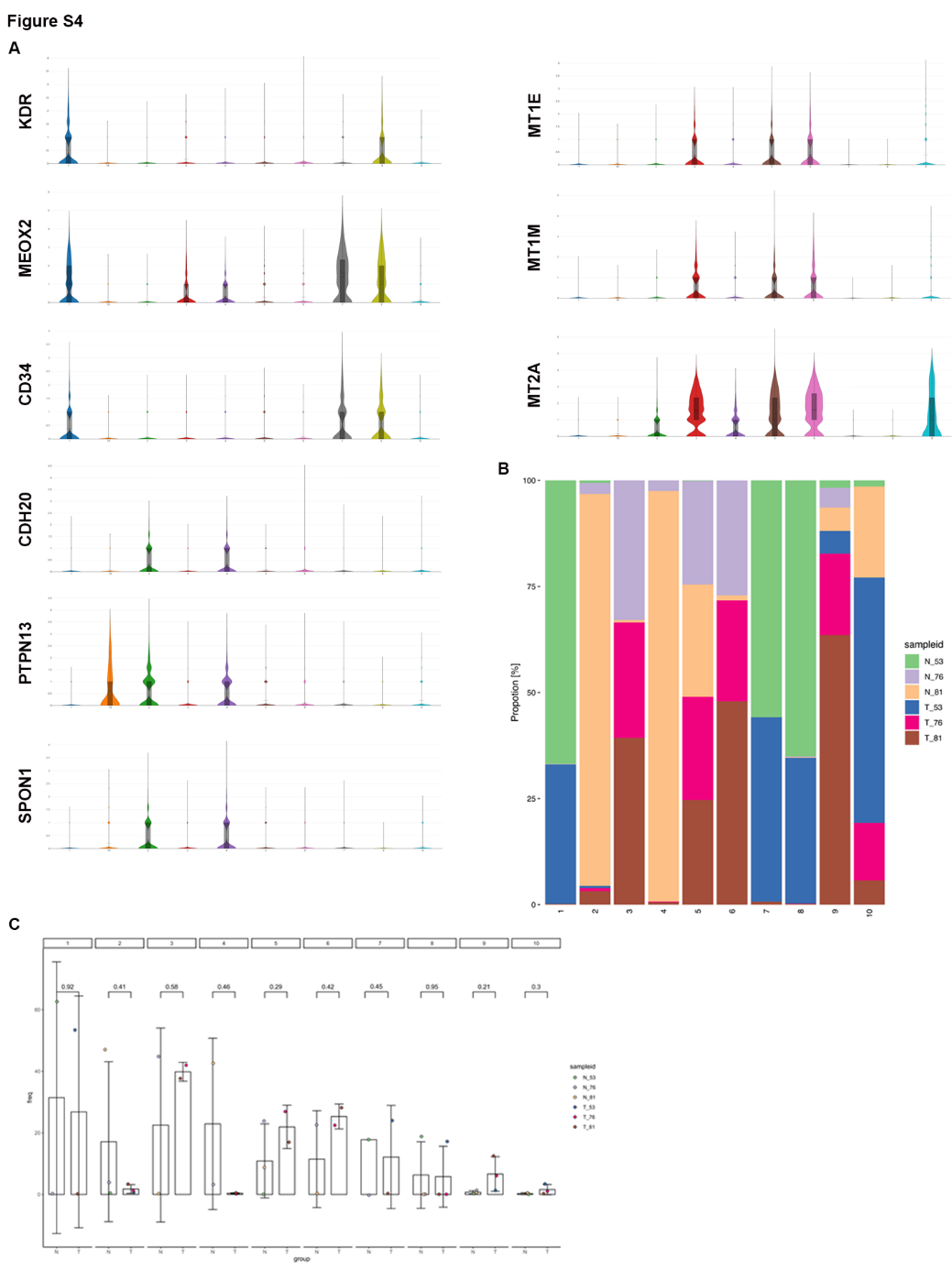


Supplementary Fig. S5. Proportion of 4 FAP clusters in each patient

A. The proportion of 4 FAP clusters in each patient

B. The average fraction (relative to the total number of FAP nuclei) of each subpopulation in N and T group


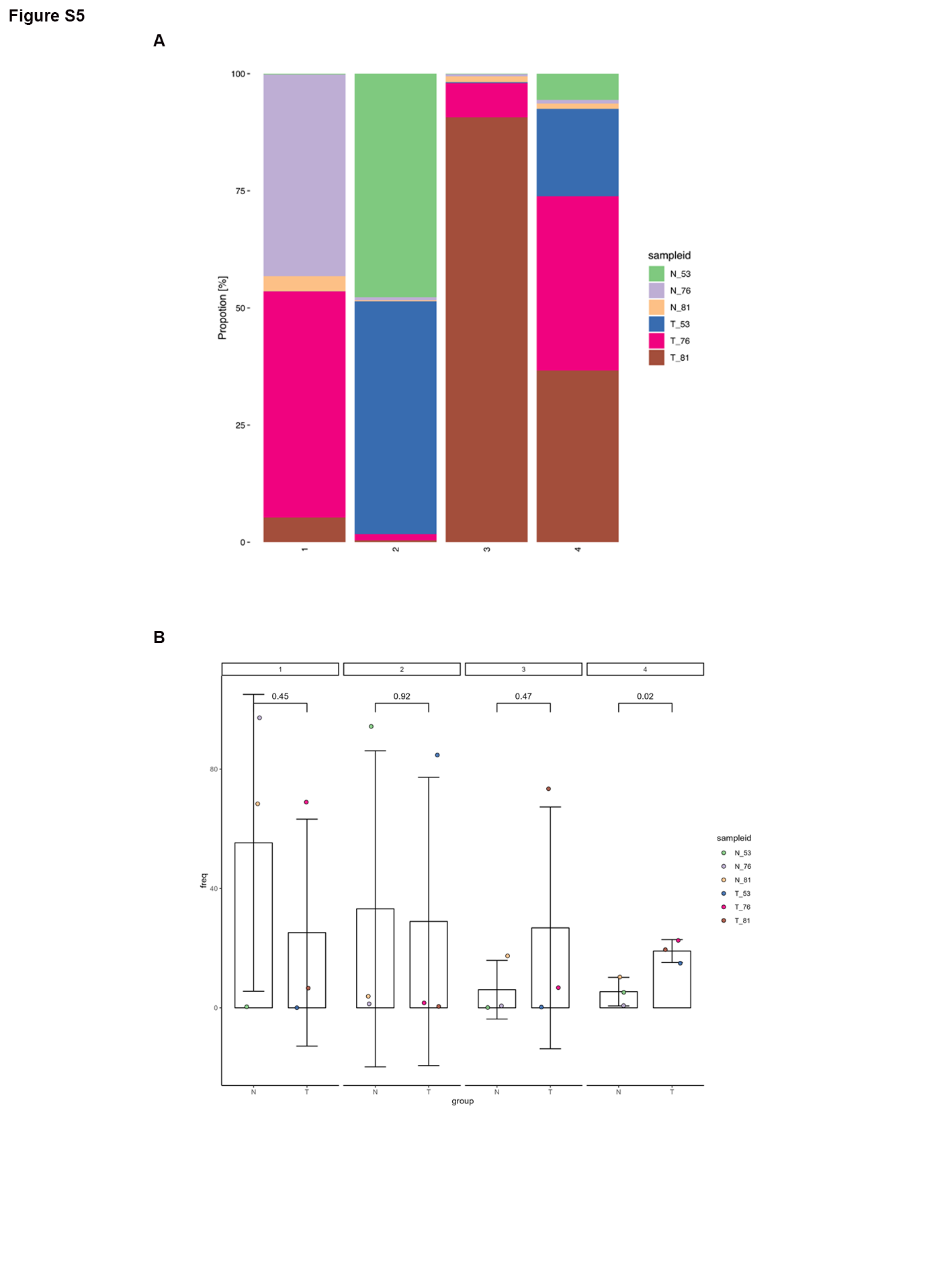


Supplementary Fig. S6. Proportion of 5 adipocyte clusters in each patient

A. The proportion of 5 adipocyte clusters in each patient

B. The average fraction (relative to the total number of adipocyte nuclei) of each subpopulation in N and T group


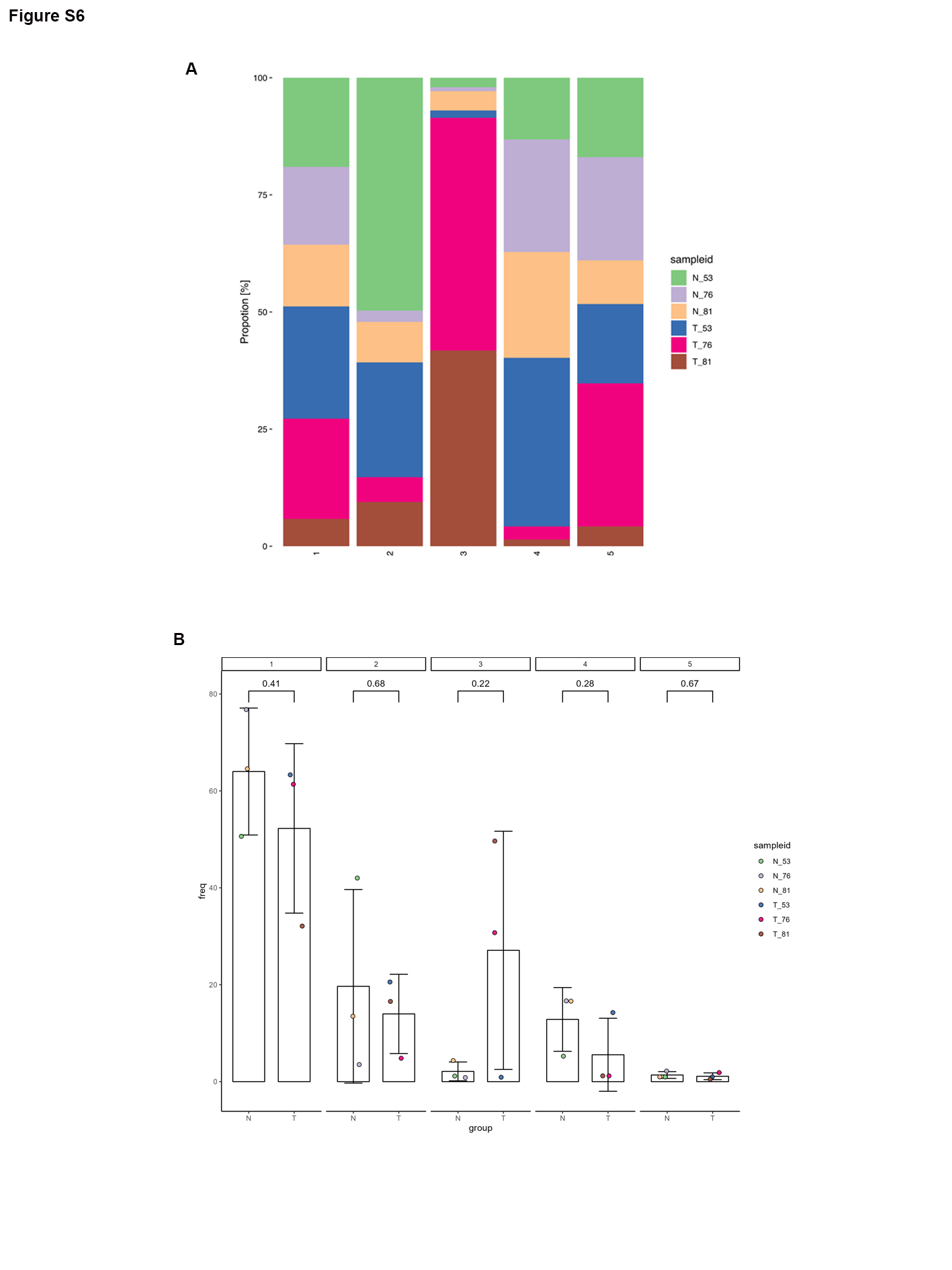


Supplementary Fig. S7. Mapping the Developmental Track of Adipocytes by Pseudotime State Transition

A. FAP1 and 5 adipocyte clusters in each patient

B. The differentiation status in each patient


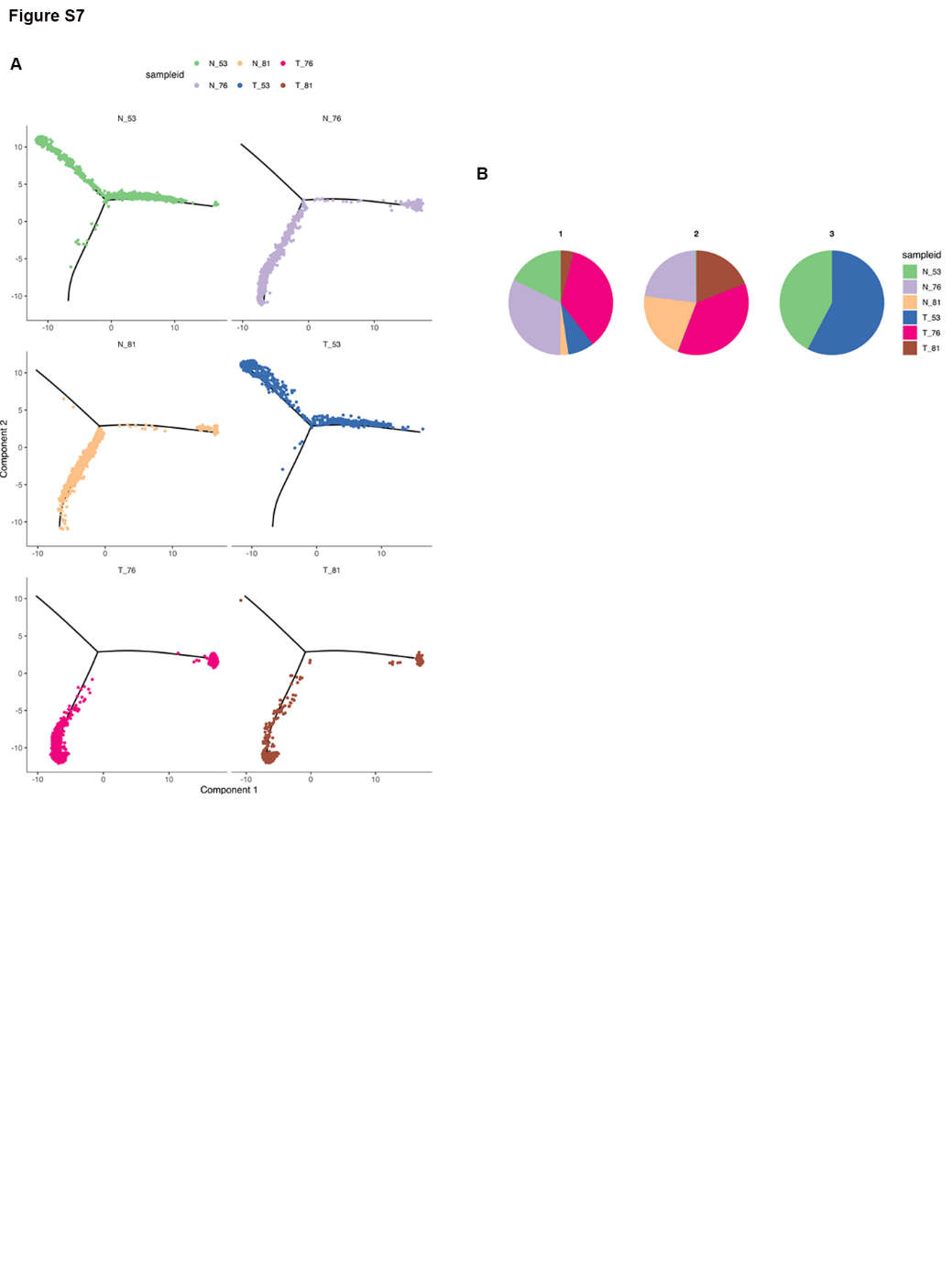


Supplementary Fig. S8. Bubble diagram showing interactions of ligands and receptors, among different cell types of each group. P values are indicated by circle size, with the scale to the right (permutation test). The color changes from blue to red, indicating a low to high communication probability.


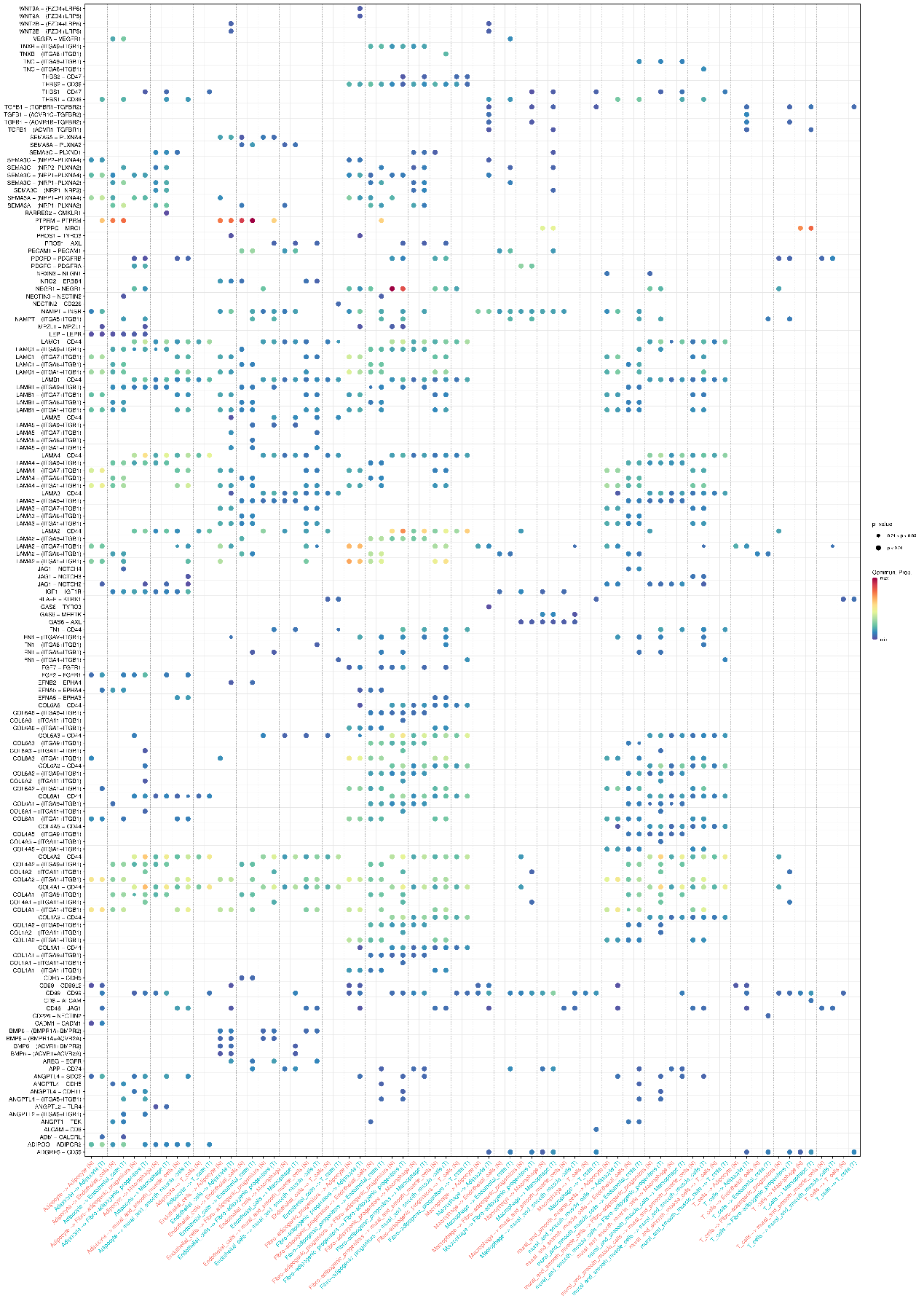

Supplement: Supplementary file 1 [file DataSheet_1.docx]
