# Supplementary material for: Diversity and heterogeneity in human breast cancer adipose tissue revealed at single-nucleus resolution
Source: Front Immunol. 2023 Apr 21;14:1158027. doi: 10.3389/fimmu.2023.1158027 (PMC10160491; doi:10.3389/fimmu.2023.1158027)
Supplement: Supplementary file 2 [file Table_1.docx]

**Table 1 Characteristics of patients included in this study**

| Patient ID | Diagnosis | Ki67 | TNM | Age | BMI | Menstruation | Subtype |
| --- | --- | --- | --- | --- | --- | --- | --- |
| Patient 81 | High grade ductal carcinoma in situ | 30%+ | pT1miNOMX | 64 | 22.3 | menopause | HER2 |
| Patient 76 | Invasive breast carcinoma | 20%+ | T1aNOMX | 61 | 21.8 | menopause | HER2 |
| Patient 53 | Invasive breast carcinoma | 40% | pT2N3aMX | 64 | 22.7 | menopause | HER2 |

**Table 2. Sequencing statistics for the three normal adipose tissue samples and three breast cancer adjacent adipose tissue samples included in this study**

| **Patient ID** | **Sample type** | **10X version*** | **Cells** | **Median UMIs/cell** | **Mean reads per cell** | **Median genes per cell** |
| --- | --- | --- | --- | --- | --- | --- |
| Patient 53 | normal adipose tissue | V3 | 12297 | 3532 | 31093 | 1901 |
| Patient 76 | normal adipose tissue | V3 | 8536 | 2873 | 43625 | 1550 |
| Patient 81 | normal adipose tissue | V3 | 11308 | 1890 | 35964 | 1302 |
| Patient 53 | Adjacent adipose tissue | V3 | 10626 | 4814 | 34528 | 2285 |
| Patient 76 | adjacent adipose tissue | V3 | 12103 | 5031 | 35460 | 2173 |
| Patient 81 | adjacent adipose tissue | V3 | 10738 | 3028 | 36282 | 1454 |

*Single-cell suspensions were converted into single-cell RNA-seq libraries using a commercially available DROP-seq protocol (10X genomics) following version 3 chemistry.
